# P-997. Successful Implementation of an Infectious Disease-Led Non-Restrictive Antimicrobial Stewardship Program in a Peruvian Hospital

**DOI:** 10.1093/ofid/ofaf695.1196

**Published:** 2026-01-11

**Authors:** Xosse Carreras, Andrea Sofia Salcedo, Sara Muñoz, Nelson Diaz, Jorge Salinas, Marisa Holubar, Jorge Alave

**Affiliations:** Universidad Peruana de Ciencias Aplicadas, Lima, Lima, Peru; Universidad Peruana de Ciencias Aplicadas, Lima, Lima, Peru; Clinica Good Hope, Lima, Lima, Peru; Universidad Peruana Union, Lima, Lima, Peru; Stanford University, Palo Alto, CA; Stanford University School of Medicine, Stanford, CA; Universidad Peruana Union, Lima, Lima, Peru

## Abstract

**Background:**

Peru reports high carbapenem and vancomycin usage with concerning resistance rates, including 60.6% third-generation cephalosporin resistance in E.coli bloodstream infections. National legislation requires antimicrobial stewardship programs (ASPs) in secondary and tertiary facilities.

**Methods:**

We implemented an infectious disease physician-led ASP at a 107-bed Peruvian hospital and report 12-month outcomes. Implementation phases included education and guideline development, prospective audit and feedback without restrictions, and data collection. The ASP targeted meropenem, vancomycin, and linezolid in medicine and ICU wards. Medical students conducted chart reviews with recommendations communicated to physicians.

**Results:**

We audited 191 records of predominantly elderly patients (median 78 years), with meropenem most prescribed (89.4%). Guideline adherence was 84.7%. Common recommendations included limiting duration (39.2%), adjusting based on cultures (31.7%), and de-escalation (18.5%)(Table1). Implementation rate was 78.8%. In medicine, consumption decreased during intervention: meropenem by 57.1% (10.5 to 4.5 DDD/100 patient-days), vancomycin by 76.7% (1.8 to 0.42), and linezolid by 50% (1.0 to 0.5)(Figure1). In ICU, reductions were 48.6% for meropenem, 50% for vancomycin, with continued post-intervention decreases(Figure2). Challenges included physician resistance, risk-averse prescribing, and suboptimal diagnostics.

**Conclusion:**

ASPs can be effectively implemented in resource-limited settings through leadership, education, and non-restrictive approaches. Success factors included collaborative methods, multimodal communication, continuous education, and transparent reporting. The post-prescription audit and feedback model maintained physician autonomy while reducing broad-spectrum antibiotic use.

**Disclosures:**

All Authors: No reported disclosures

